# Protective effects of three remote ischemic conditioning procedures against renal ischemic/reperfusion injury in rat kidneys: a comparative study

**DOI:** 10.1007/s11845-014-1227-8

**Published:** 2014-11-15

**Authors:** H. Jiang, R. Chen, S. Xue, H. Zhu, Xiaolei Sun, Xiaoqing Sun

**Affiliations:** 1 The Department of Urology, The Affiliated Hospital of Xuzhou Medical College, 99 Huaihai West Road, Xuzhou, 221000 Jiangsu China; 2 The Urinary Laboratory, The Affiliated Hospital of Xuzhou Medical College, 99 Huaihai West Road, Xuzhou, 221000 Jiangsu China

**Keywords:** Remote ischemic perconditioning, Remote ischemic postconditioning, Kidney, Ischemic reperfusion injury

## Abstract

**Background:**

Remote ischemic perconditioning (RIPerC), remote ischemic postconditioning (RIPostC), and remote ischemic perconditioning + postconditioning (RIPerC + RIPostC) protect against renal ischemia reperfusion injury (IRI). However, the most beneficial approach among these is not known.

**Aims:**

To compare the protective effects and study the mechanisms of three different remote ischemic conditioning in preventing IRI in the rat kidney.

**Methods:**

Fifty healthy adult male Sprague–Dawley rats were randomly assigned to five groups: sham, IRI, RIPerC, RIPostC, and RIPerC + RIPostC. Right nephrectomy was performed initially in all rats. IRI was induced by occluding the left renal artery for 60 min, followed by reperfusion for 24 h. RIPerC, RIPostC, and RIPerC + RIPostC were induced with 5-min ischemia/reperfusion (I/R) cycles using a tourniquet on the right hind limb.

**Results:**

The IRI group showed significant serologic evidence of renal injury compared to the sham group (*P* < 0.05). The RIPerC, RIPostC, and RIperC + RIpostC groups displayed significantly lower levels of renal dysfunction than the IRI group (*P* < 0.05). Superoxide dismutase (SOD) levels were significantly lower in the IRI group than in the sham group (*P* = 0.003), but were significantly less depressed in the RIPerC, RIPostC, and RIperC + RIpostC groups (*P* < 0.05). The IRI group displayed more severe renal tubular injury than the RIPerC, RIPostC, and RIPerC + RIPostC groups (*P* < 0.05).

**Conclusion:**

All three remote ischemic conditioning showed similar therapeutic potential for preventing renal IRI. The RIPerC + RIPostC protocol did not show an additive effect from the combination of preconditioning and postconditioning. The protective mechanism may be due to the stimulation of endogenous antioxidant activity by transient limb ischemia–reperfusion.

## Introduction

Ischemia–reperfusion injury (IRI) occurs when blood supply to a tissue is temporarily interrupted. When blood flow is restored reperfusion paradoxically induces more severe tissue injury [1, 2]. Renal warm-IRI occurs in clinical practice and is a consequence of systemic hypoperfusion with subsequent circulatory resuscitation. Local nephritic hypoperfusion after aortic cross-clamping or renal transplantation also causes IRI to the kidney.

Several recent trials have shown that remote ischemic conditioning (RIC) has a powerful protective effect in limiting nephritic IRI [3]. RIC is accomplished with brief nonlethal cycles of ischemia and reperfusion of an arm or leg. These cycles may be applied before (preconditioning), during (perconditioning) [4], or after (postconditioning) [5] prolonged ischemia of a distant organ [6, 7]. Some studies have found that RIC is a straightforward, inexpensive, non-invasive, and powerful means of preventing nephritic IRI during surgery or organ transplantation [3, 8, 9].

In previous studies, remote ischemic postconditioning (RIPostC) and perconditioning (RIPerC) have provided practical methods for protecting the kidneys against IRI [3], but their combined effects and mechanism have not been studied in detail.

In the present study, we conducted a randomized trial on rats in which we induced IRI. To augment the protective effect of RIC, RIC was induced through lower limb ischemia, rather than upper limb ischemia, and RIPerC was combined with RIPostC (RIPerC + RIPostC). Renal IRI was assessed by measuring levels of serum creatinine (SCr) and blood urea nitrogen (BUN). We also histologically assessed the degree of renal tubular injury, and measured myeloperoxidase (MPO) activity superoxide dismutase (SOD) activity and malondialdehyde (MDA) content.

## Materials and methods

### Animals

Eighteen-week-old male Sprague–Dawley rats, weighing between 200 and 250 g (Experimental Animal Center, Xuzhou Medical College, Xuzhou, Jiangsu province, China) were studied. The animal research study protocol was in compliance with the Guide for the Care and Use of Laboratory Animals published by the National Institutes of Health (NIH Pub. No. 85–23, revised 1996) and approved by the Animal Care Committee of the Affiliated Hospital of Xuzhou Medical College, Xuzhou Medical College. All rats were acclimatized with free access to food and water in a 22–27 °C environment for 2 weeks prior to the experiments.

### Experimental design and surgical procedure

**Fig. 1:**
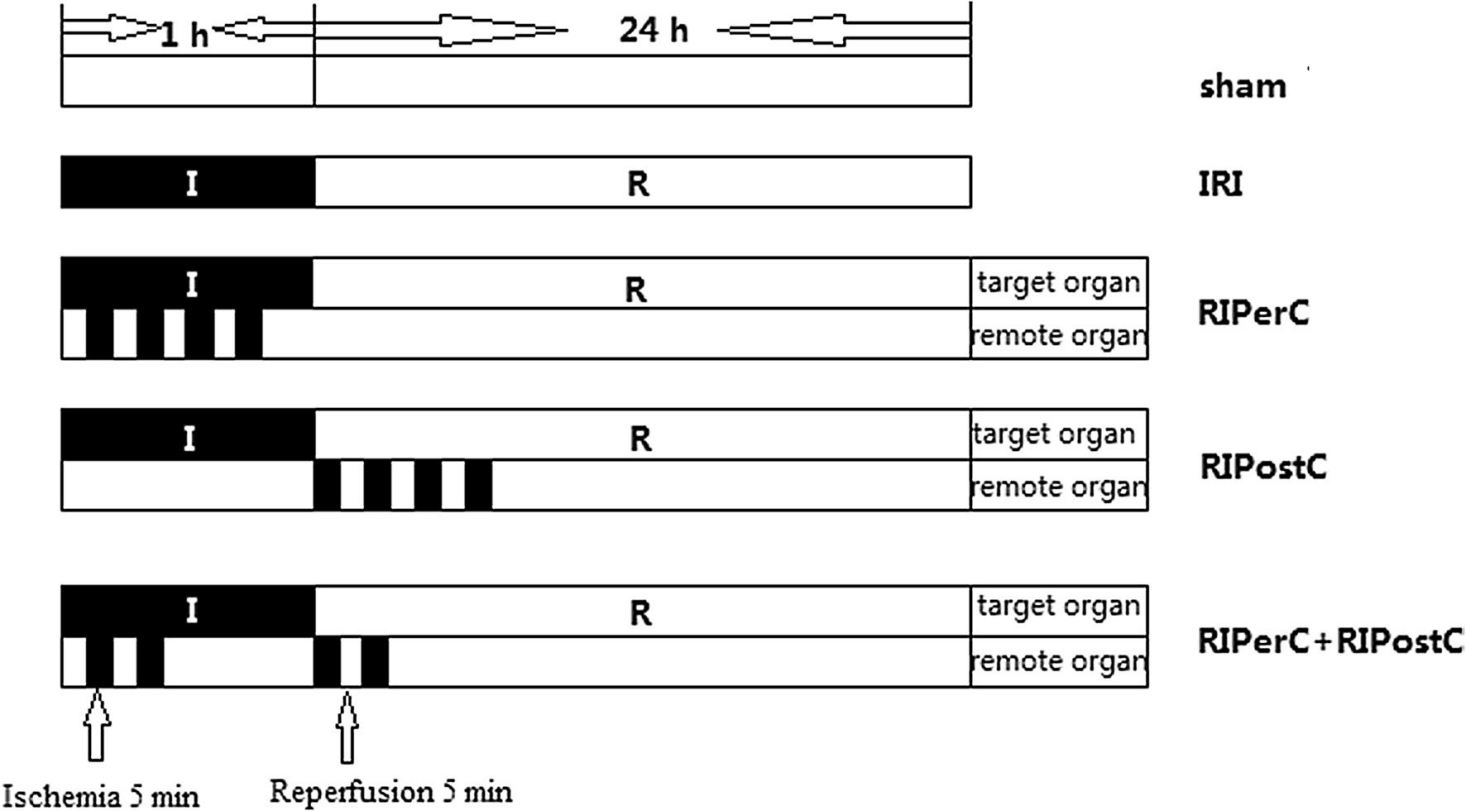
Experimental protocols and grouping of the animals

All rats were fasted 12 h before surgery. Surgical procedures were performed with the rats under sodium pentobarbital anaesthesia (40 mg/kg; I.P.). The RIC stimulus was delivered via tourniquet blockage of blood flow to the right hind limb for cycles of 5-min occlusion followed by 5-min resumption of blood flow. Fifty rats were randomly allocated to each of five experimental groups; three rats were excluded because of anaesthetic or surgical complications. All animals underwent right nephrectomy. In the IRI group (*n* = 10), the left renal artery was occluded for 60 min with a nontraumatic vascular clip, followed by 24 h of reperfusion. In the sham group (*n* = 10), all the above surgical procedures were performed, except that IRI was not induced. In the RIPerC group (*n* = 8), four cycles of 5 min of ischemia followed by 5 min of reperfusion were performed on the right hind limb during renal ischemia and before renal reperfusion. In the RIPostC group (*n* = 9), four cycles of ischemia/reperfusion of the right lower limb were performed immediately after restoring blood flow to the kidney. In the RIPerC + RIPostC group (*n* = 10), two cycles of ischemia/reperfusion were performed during renal ischemia before renal reperfusion, and two similar cycles were performed immediately upon restoring blood flow to the kidney (Fig. 1).

### Measurement of SCr and BUN concentrations

At the end of the 24-h reperfusion period, plasma samples were collected, and SCr and BUN levels were determined using commercially available colorimetric methods, according to the manufacturer’s instructions.

### Measurement of MPO, MDA, and SOD in renal tissue

Renal tissue samples were collected and 10 % homogenate samples were prepared. MPO activity, MDA content, and SOD activity were measured in the 10 % homogenates by colorimetric methods using commercially available kits, according to the manufacturer’s instructions.

### Histology

In each rat, the left kidney was removed under fully maintained anaesthesia. Animals were sacrificed only after removal of the left kidney. After removal, the kidney was bisected along the non-hilar axis and was fixed in 10 % phosphate-buffered formalin. The tissues were subsequently embedded in paraffin, sectioned, stained with hematoxylin and eosin, and were analyzed. Renal damaged was histologically graded using the established by accepted grading system described by Jablonski et al. [10]. Kidney injury was scored by a single pathologist (X.L.S.) as the percentage of damaged tubules in the corticomedullary junction. Criteria for kidney injury included tubular necrosis, cast formation, loss of the brush border, tubular dilatation and immune cell infiltration. Scoring for each category was as follows: 0 for no change; 1 for <10 %; 2 for 10–20 %; 3 for 21–30 %; 4 for >30 % area change. Scores for all categories were added for the final injury score.

### Calculations and statistics

Graph Pad Prism 5 (Graph Pad Inc., La Jolla, CA) was used to analyze and present data. Differences between groups were analyzed using a paired parametric t test or a one-way ANOVA test. Values are expressed as the mean ± SEM. A *P* value <0.05 was considered statistically significant.

## Results

### Functional assessment

**Fig. 2:**
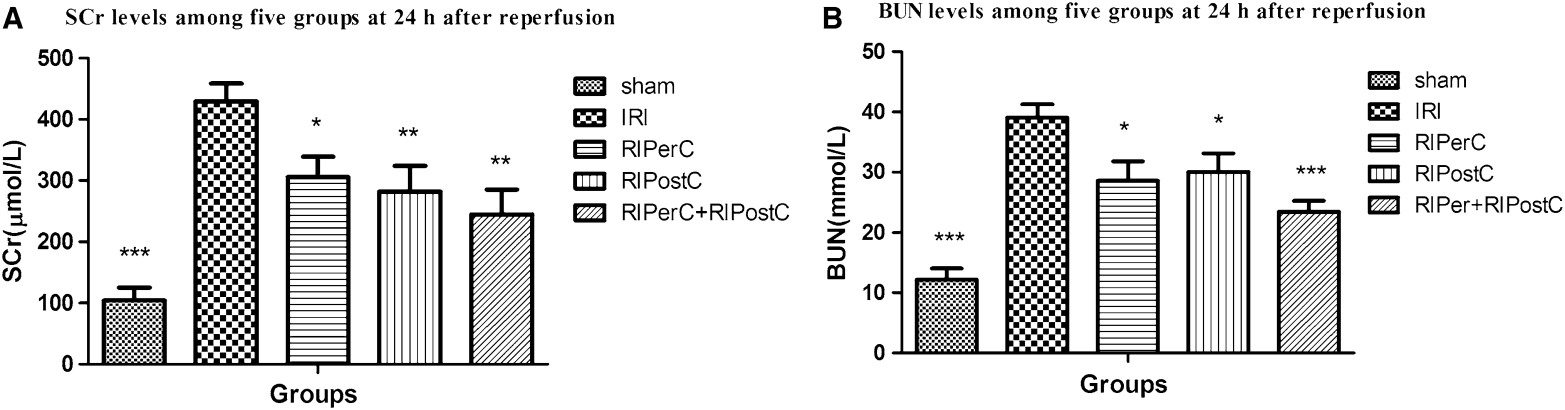
**a** and **b**, respectively demonstrate that SCr and BUN concentrations in the IRI, RIPerC, RIPostC, and RIPerC + RIPostC groups are significantly higher than those in the sham group. In addition, the IRI group shows higher SCr and BUN concentrations than the RIPerC, RIPostC, and RIPerC + RIPostC groups. (*** *P* < 0.001, ** *P* < 0.01, * *P* < 0.05 vs. IRI group)

As shown in Fig. 2a, b, the levels of SCr and BUN in the IRI, RIPerC, RIPostC and RIPerC + RIPostC groups were significantly higher than seen in the sham group (*P* < 0.001). In addition, the IRI group showed higher levels of SCr and BUN than the RIPerC, RIPostC, and RIPerC + RIPostC groups. There was no significant difference in SCr and BUN levels among the RIPerC, RIPostC, and RIPerC + RIPostC groups at 24 h after reperfusion (*P* > 0.05). These results indicate that the renal IRI induced by a 60-min period of ischemia can be limited to some degree using RIC.

### SOD activity, MPO activity, and MDA content

**Fig. 3:**
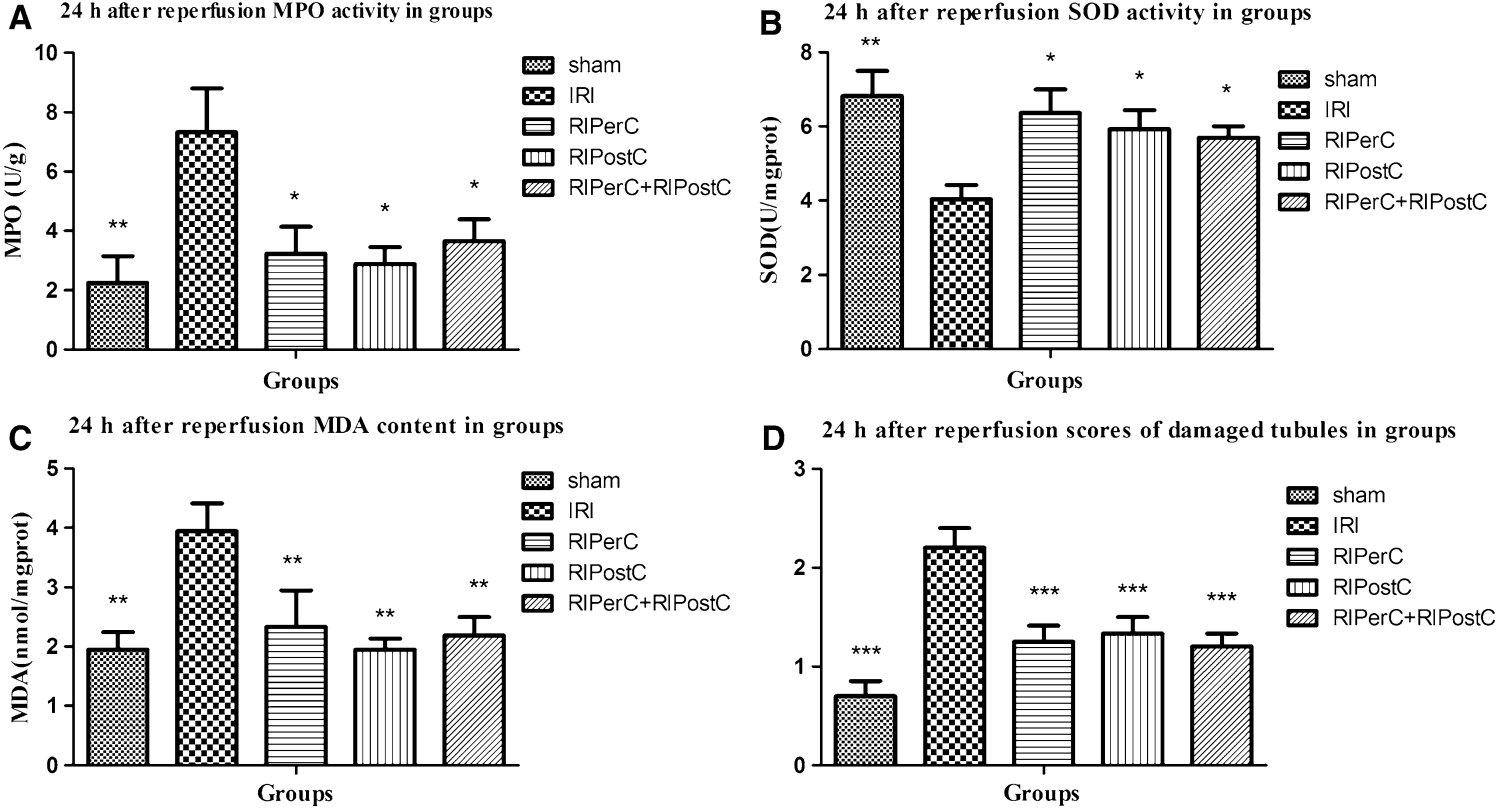
**a** and **c**, respectively, demonstrate that MPO activity and MDA content levels are significantly elevated in the IRI group compared to those in the sham group. All of the RIC groups (RIPerC, RIPostC, and RIPerC + RIPostC) had a lesser elevation of MPO activity and MDA content. **c** Demonstrates that SOD activity significantly decreased in the IRI group compared to that in the sham group, but did not significantly decrease in the RIC groups (RIPerC, RIPostC, and RIPerC + RIPostC groups). **d** Shows that the tubular damage score significantly decreased in the RIC groups (RIPerC, RIPostC, and RIPerC + RIPostC groups) compared to that the IRI group. (*** *P* < 0.001, ** *P* < 0.01, * *P* < 0.05 vs. IRI group)

MPO activity and MDA content levels were significantly elevated in the IRI group compared to the sham group. All of the RIC groups (RIPerC, RIPostC, and RIPerC + RIPostC) had a less remarkable elevation of MPO activity and MDA content (*P* < 0.05, Fig. 3a, c). SOD activity significantly decreased in the IRI group compared to that in the sham group, but did not significantly decrease in the RIC groups (*P* < 0.05, Fig. 3b). SOD activity, MPO activity, and MDA content were not significantly different between the RIPerC, RIPostC and RIPerC + RIPostC groups. (*P* > 0.05, Fig. 3).

### Histological assessment

**Fig. 4:**
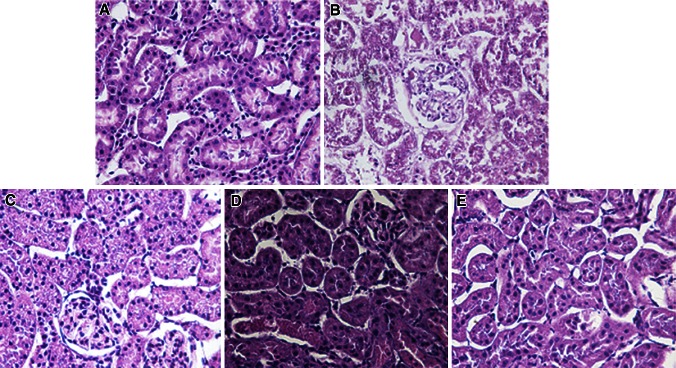
Tissue sections stained with hematoxylin and eosin (H&E) ×40. **a** Sham operation (sham group): no abnormalities; **b** untreated IRI (IRI group): moderate-to-severe tubular cell necrosis, tubular dilation, intratubular cell detachment, interstitial oedema, and interstitial cellular infiltration; **c**, **d**, **e** RIPerC, RIPostC, and RIPerC + RIPostC treatment (RIPerC, RIPostC, and RIPerC + RIPostC groups): the degree of renal graft injury clearly less severe than that in the IRI group (B)

According to the well-known grading system established by Jablonski et al. the extent of renal tubular damage in the study groups is described in detail in Table 1. The RIPerC, RIPostC, and RIPerC + RIPostC groups showed ischemic tubulointerstitial abnormalities, that were clearly less prominent than those seen in the IRI group, which displayed moderate-to-severe ischemic-characteristic tubulointerstitial lesions (*P* < 0.05, Figs. 3d, 4). A significant difference was not seen among the RIPerC, RIPostC, and RIPerC + RIPostC groups at 24 h after kidney reperfusion.

**Table 1:** Renal tubular damage scores in the five groups

Groups	Degree of damage				
	No change (*n*)	Minimal (<10 %) (*n*)	Mild (11–20 %) (*n*)	Moderate (21–30 %) (*n*)	Severe (>30 %) (*n*)
Sham (*n* = 10)	3	7	0	0	0
IRI (*n* = 10)	0	1	6	3	0
RIPerC (*n* = 8)	0	6	2	0	0
RIPostC (*n* = 9)	0	6	3	0	0
RIPerC + RIPostC (*n* = 10)	0	8	2	0	0

## Discussion

The present study shows that RIPerC + RIPostC was as effective as RIPerC or RIPostC in decreasing renal reperfusion injury in a rat model of IRI. However, the RIPerC + RIPostC combination did not result in superior outcomes to either RIPerC or RIPostC alone.

The renal IRI protective effect of RIPerC and RIPostC was first reported by Kadkhodaee et al. [3] in 2011. In a rat model of nephritic IRI, four episodes of 5-min ischemia followed by 5-min reperfusion of the left femoral artery were applied during renal ischemia before reperfusion, or after ischemia at the time of restoration of blood flow to the kidney.

We included five experimental groups to investigate whether RIC could protect the kidney from ischemic injury. Indeed, the IRI group demonstrated significantly more serologic and histological evidence of renal damage than the sham group, verifying the induction of ischemia/reperfusion injury. Additionally, SCr and BUN levels in the RIPerC, RIPostC and RIPerC + RIPostC groups were significantly lower than those in the IRI group (*P* < 0.05), demonstrating the protective effect of RIC. There was no significant difference in SCr or BUN levels between the three RIC groups (*P* > 0.05).

MPO accounts for about 5 % of the dry cell weight of neutrophils. The level of MPO activity in renal tissue represents the quantitative expression of neutrophil activity and infiltration into the kidneys. MPO dose not induce cell apoptosis, but in 1966, Yang et al. demonstrated that the release of proteinase 3 (PR3) and elastase by activated neutrophils during acute inflammation, may result in vascular damage by causing endothelial cell apoptosis [10, 11]. In other words, MPO activity may indirectly reflect the extent of renal tubular epithelial cell apoptosis [12]. The IRI, RIPerC, RIPostC, and RIPerC + RIPostC groups all showed significantly higher MPO activity than the sham group (*P* < 0.05). This indicates that after ischemia–reperfusion, a large number of neutrophils are activated, and these infiltrate into the local ischemic tissues. MPO activities in the RIPerC, RIPostC, and RIPerC + RIPostC groups were lower than those in the IRI group (*P* < 0.05), once again supporting the protective effect of RIC. The degree of nephritic tubular injury in the RIPerC, RIPostC, and RIPerC + RIPostC groups was less prominent than that seen in the IRI group (*P* < 0.05). These results suggest that the RIPerC, RIPostC, and RIPerC + RIPostC groups experienced less renal tubular damage through a reduction of neutrophil accumulation and renal tubular epithelial cell apoptosis in ischemic renal ischemic tissue.

MDA is the main metabolite of lipid peroxidation within the body, and elevated levels indirectly reflect a higher degree of cell damage in the body. SOD plays a key role in the body’s oxidation and antioxidative balance; this enzyme protects cells from damage by scavenging the superoxide anion radical (O2-). SOD activity indirectly reflects the level of free radical scavenging activity within the body, and the level of MDA indirectly reflects the severity of cellular injury from free radical attack. The IRI, RIPerC, RIPostC, and RIPerC + RIPostC groups all demonstrated higher renal tissue MDA content than the sham surgery group. SOD activity was significantly lower in the IRI group than SOD activities in the RIPerC, RIPostC, and RIPerC + RIPostC treatment groups (*P* < 0.05). The RIPerC, RIPostC, and RIPerC + RIPostC groups were able to maintain a higher SOD activity than the IRI group. This enables the destruction of more oxygen-free radicals, and reduces oxygen-free radical-mediated lipid peroxidation. The higher SOD activity in the RIC groups leads to a reduction in the degree of renal IRI and may account for a significant portion of RIC’s protective effect.

Our results indicated that RIC reduced the intensity of renal inflammation intensity, protecting the kidneys from IRI to some extent. There was no significant difference between the three RIC treatment groups. These three different approaches likely function via the same mechanism of action to reduce renal IRI. In clinical studies, the protective role of RIC in preventing IRI has been controversial. Ravlo et al. [13], Wu et al. [14] and Søndergaard et al. [8] observed that RIC can protect kidney transplant patients from renal IRI. Huang et al. also observed similar protective effects in kidney resection, but Chen et al. [15] found no improvement in renal function when RIC was used in living donor kidney transplantation. Further studies are necessary to determine the optimal techniques and indications for RIC in the future, especially with regard to clinical trials.

## Conclusion

We have demonstrated the protective effects of RIC in limiting IRI in the rat kidney. RIPerC, RIPostC, and RIPerC + RIPostC treatments were all equally beneficial, and possibly exerted their effects by enhancing antioxidant activity and decreasing inflammation in the injured renal tissues. The RIPerC + RIPostC protocol did not show an additive effect from the combination of perconditioning and postconditioning. This may be because each protocol consisted of 4 remote I/R cycles, which in the RIPerC + RIPostC protocol were split evenly between perconditioning and postconditioning.
